# Triggered: Discovery of Neurocysticercosis Following Self-Administered Albendazole

**DOI:** 10.7759/cureus.43746

**Published:** 2023-08-19

**Authors:** William R Southall, Madelaine S Southall, Mohamad B Aldaas, Vishwanath Sagi, Phani V Akella

**Affiliations:** 1 Neurology, University of Louisville School of Medicine, Louisville, USA; 2 Pediatrics, University of Louisville School of Medicine, Louisville, USA; 3 Infectious Diseases, University of Louisville School of Medicine, Louisville, USA; 4 Internal Medicine, Olathe Medical Center, Olathe, USA

**Keywords:** corticosteroids, antiparasitics, praziquantel, albendazole, taeniasis, multiple parenchymal neurocysticercosis, ncc, neurocysticercosis

## Abstract

A 25-year-old man with no medical history presented with a seizure one month after taking a self-administered dose of albendazole. Magnetic resonance imaging (MRI) of the brain revealed multiple ring-enhancing lesions, and the workup confirmed neurocysticercosis (NCC). Treatment with antiparasitics was delayed due to concern for worsening symptoms from the presence of cysts in the midbrain and hippocampus. The balance between treating NCC and limiting cerebral inflammation is delicate and relies on judgment from a multispecialty clinical team. In this case, corticosteroids and antiepileptics alone prevented additional seizures but failed to reduce the overall inflammation of cysts and the progression of the disease. Evidence of new cysts on MRI at week 13 from the onset of symptoms was evidence of an acute, evolving infectious process. Treatment with albendazole and praziquantel was initiated at 13 weeks from the onset of symptoms, and by 31 weeks, nearly all cysts had resolved with minimal residual inflammation.

## Introduction

The tapeworm, *Taenia solium*, most commonly infects humans who live in endemic areas such as Latin America, Asia, and Sub-Saharan Africa. The life cycle of *T. solium *is complex and involves humans as the definitive host and swine as the most common intermediate host. Humans become infected with the adult form of the tapeworm, a condition known as taeniasis, by ingesting undercooked pork containing *T. solium* larval cysts. These cysts hatch in the lumen of human intestines and grow into adult tapeworms that shed eggs in human feces. Swine acts as the intermediate host by ingesting *T. solium* eggs within human feces. Once inside the intestines of swine, the life cycle is completed when the eggs hatch into the larval form of *T. solium* and encyst within the skeletal muscle and other tissues [1].

Cysticercosis occurs when *T. solium* eggs are ingested via fecal-oral contamination by humans rather than swine. Humans become an accidental intermediate host and the larvae spread to skeletal muscles and other tissues to form cysts. This process can occur by consuming food contaminated by human feces or by auto-infection. Commonly, cysts migrate to the central nervous system (CNS) leading to a condition known as neurocysticercosis (NCC). Symptoms of NCC vary depending on the location, number of cysts, and the body’s inflammatory response to them. In symptomatic patients, seizures are the most common manifestation (80% of symptomatic cases), but focal neurological deficits, intracranial hypertension, hydrocephalus, chronic meningitis, and other abnormalities may occur [1,2].

Once *T. solium* eggs are consumed, they must pass through the intestinal wall of the host and evade the host's immune system to establish themselves as cysts [3]. Most larvae fail to effectively evade the immune system, making the sheer number of eggs consumed by the host an important factor in determining the severity of the disease [4]. The degeneration of *T. solium* cysts within the brain is a continuum categorized into four groups based on neuroimaging: vesicular, colloidal, granular-nodular, and calcified [5,6]. The vesicular stage corresponds to cysts that have achieved immune tolerance. Vesicular cysts have a scolex that is often visible on magnetic resonance imaging (MRI), but they generally do not have perilesional edema and thus are non-contrast enhancing [6,7]. The colloidal stage corresponds to the beginning of initial host immune recognition that leads to perilesional inflammation and contrast enhancement. The granular-nodular stage represents further damage to the cyst where fluid contents within the cyst are no longer discernible on imaging. It appears as nodules surrounded by T2 hyperintense rims of gliosis and remains contrast-enhancing on MRI. Calcified lesions represent the destroyed remnants of cysts and appear as non-contrast enhancing lesions best seen with computed tomography (CT). Calcified lesions are not well visualized by MRI [6,7].

The degeneration of cysts and progression through the four stages begins once the immune system recognizes the cysts, but what begins this process is unknown. The latent period between infection and symptoms can range from months to decades [8]. We describe a rare report of active and evolving NCC in the United States in which the onset of symptoms and degeneration of cysts was triggered by a single dose of self-administered antiparasitic treatment. We hypothesize that this single dose of antiparasitic therapy disrupted the immune tolerance state of the cysts, leading to the presenting symptoms.

## Case presentation

A 25-year-old Asian male without significant past medical history presented to the emergency room after an episode of loss of consciousness and tongue laceration. The event was unwitnessed, lasted 30 minutes, and was preceded by one month of bilateral upper and lower extremity weakness and two days of holocranial headache, sweating, and malaise. One month prior, the patient noticed a large flatworm in his stool for which he took a single dose of albendazole from an undisclosed source. The patient immigrated to the United States from India four years prior and had been living in California and Kentucky. The patient denied eating pork until he moved to the United States in 2018.

**Figure 1:**
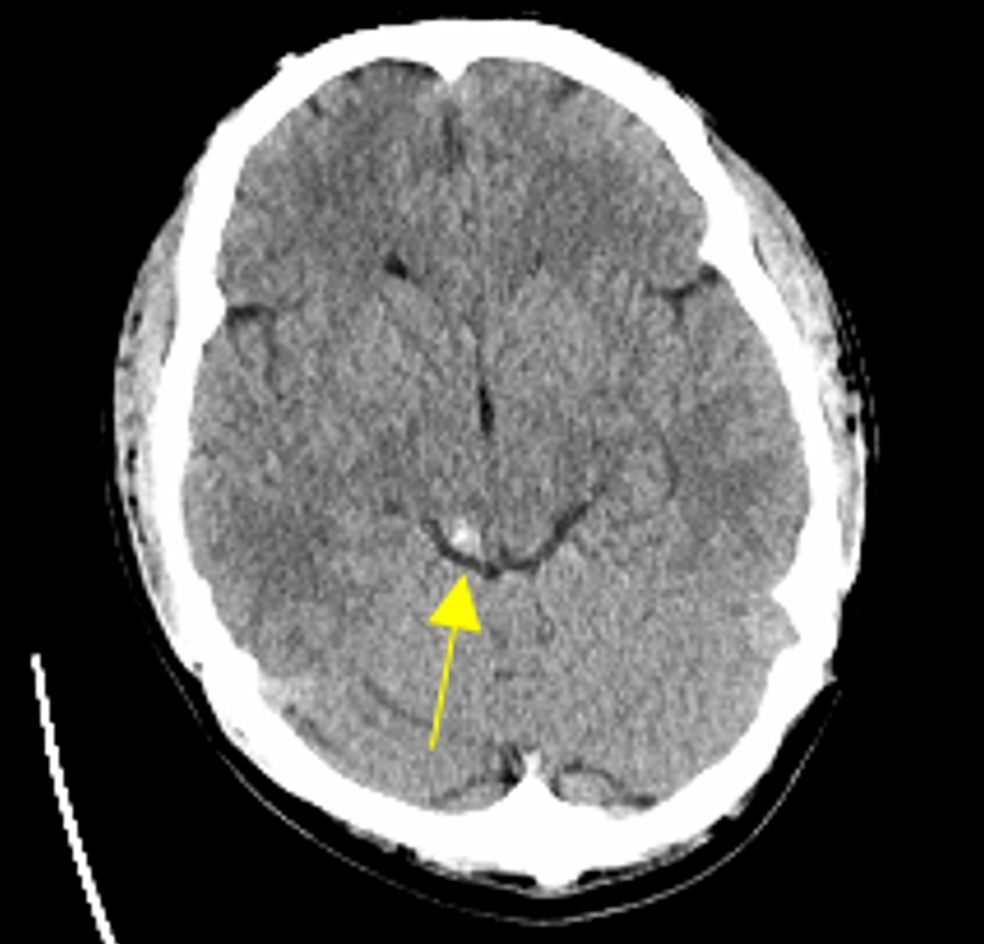
CT of the head at the onset of symptoms A 5 mm hyperdensity in the right posterior midbrain is shown.

**Figure 2:**
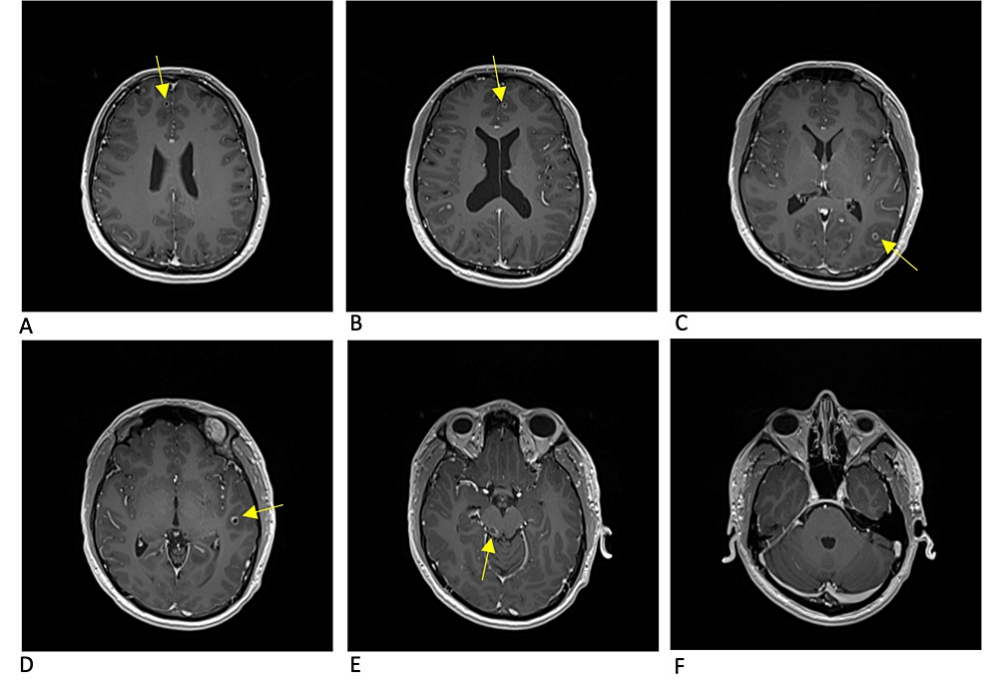
MRI brain T1 axial post-contrast sequence at the onset of symptoms There are multiple bilateral ring-enhancing lesions as denoted by arrows. Multiple cystic lesions can be found in the anterior right and left frontal lobes (panes A and B). Cystic lesions with increased amounts of perilesional edema can be found in the posterior left parietal lobe and left temporal lobe (panes C and D). The lesion located in the right posterior midbrain is the largest of all cysts with a significant amount of perilesional edema (pane E). Lesions depicted are in the colloidal to granular-nodular stages. Note: Pane F demonstrates the absence of cysts in the cerebellum.

**Figure 3:**
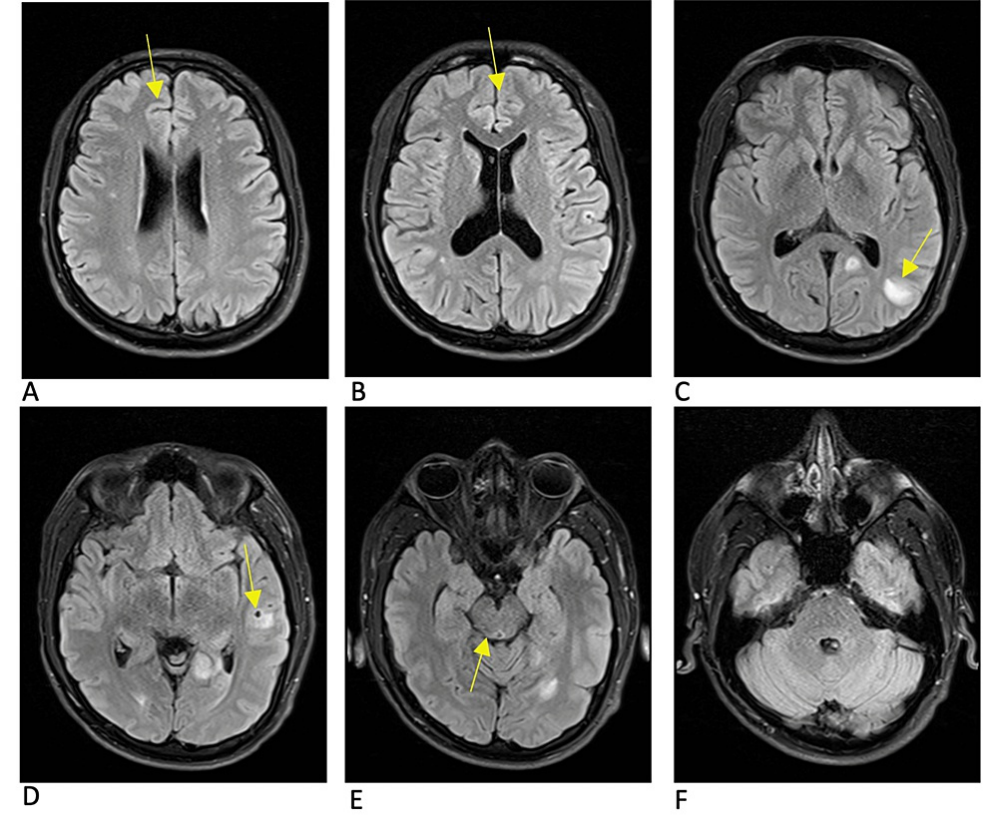
MRI brain T2 FLAIR sequence at the onset of symptoms Multiple bilateral inflammatory lesions are denoted by arrows. The cysts in the anterior right and left frontal lobes (panes A and B) are associated with much less inflammation when compared to the posterior left parietal lesion and the left temporal lesion (panes C and D). The right posterior midbrain lesion demonstrates less T2 fluid-attenuated inversion recovery (FLAIR) signal than would be expected given its size (pane E). There are multiple areas of inflammation seen on T2 FLAIR that do not correlate with a lesion on the T1 post-contrast sequence demonstrating potential additional cysts.

Initial exam revealed an awake, alert, and afebrile male not in acute distress with normal vital signs. His neurological and ophthalmological exam showed no abnormalities. A comprehensive metabolic panel, complete blood count, urinalysis, and drug screen were unremarkable. Further workup revealed normal troponin levels, normal electrocardiogram, normal echocardiogram, and normal CT angiogram. A CT scan of the head without contrast revealed a 5 mm hyperdensity in the right posterior midbrain (Figure 1) and nonspecific small areas of edema in the left temporal and parietal lobes. MRI of the brain with and without contrast revealed multiple supratentorial predominantly juxtacortical ring-enhancing lesions with surrounding vasogenic edema, including a lesion in the right posterior midbrain (Figures 2, 3). A seizure was suspected based on symptoms and radiographic findings. Levetiracetam 500 mg twice a day was started for seizure prophylaxis and subsequent electroencephalogram showed no abnormalities.

Due to multiple ring-enhancing lesions on imaging and a history of parasites in the stool, NCC was atop the differential diagnoses that also included tuberculosis, histoplasmosis, toxoplasma, atypical bacterial abscess, and metastatic cancer. Empiric coverage was started with intravenous vancomycin and ceftriaxone. Intravenous dexamethasone (4 mg every six hours) was started for perilesional cerebral edema.

Cerebrospinal fluid (CSF) analysis showed an elevated white blood cell count of 152/μL (56% lymphocytes), elevated protein of 49 mg/dL, and glucose of 55 mg/dL (serum glucose was 113 mg/dl). Opening pressure was normal (20 cm H2O). The patient tested negative for HIV, toxoplasma, and *Cryptococcus*. CSF fungal, bacterial, and acid-fast bacilli cultures showed no growth. The meningitis encephalitis polymerase chain reaction (PCR) panel of the CSF was negative. Stool examination was negative for ova and parasites. QuantiFERON Gold and *Strongyloides* serologies were negative. Enzyme-linked immunotransfer blot of the serum for *T. solium* antibodies was positive and confirmed the suspected diagnosis of NCC.

Due to concern for multiple parenchymal lesions with edema and the risk of triggering a widespread central nervous system (CNS) immune response, antiparasitic therapy was deferred at that time. The patient was discharged home on oral levetiracetam 500 mg twice a day and dexamethasone was tapered to 1 mg once a day. Levetiracetam was subsequently switched to oxcarbazepine 600 mg twice a day due to irritability.

**Figure 4:**
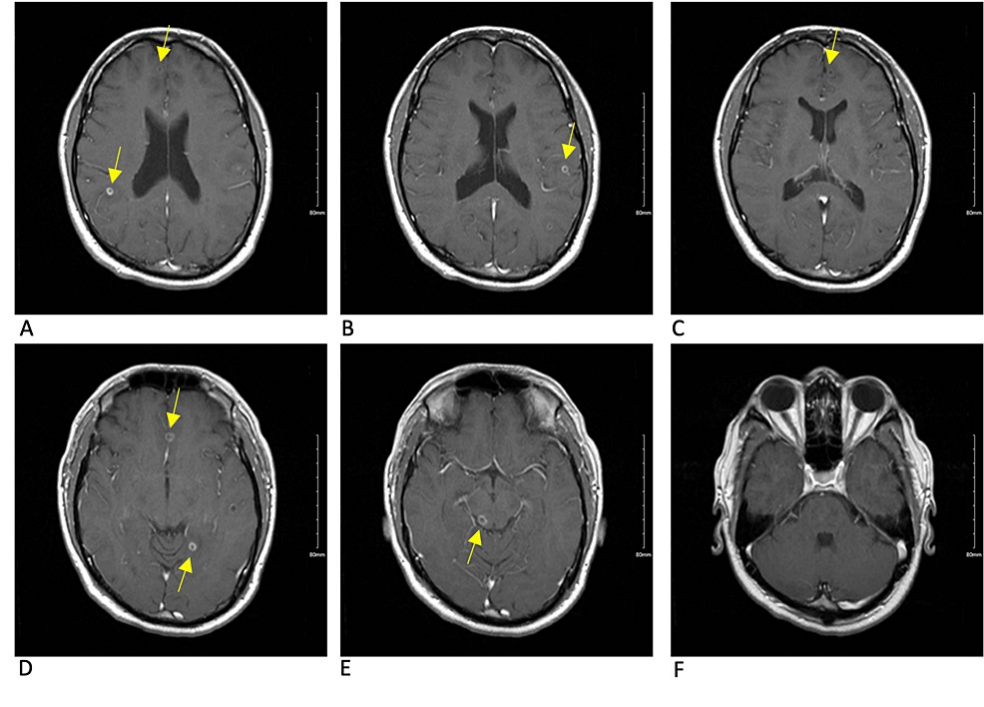
MRI brain T1 axial post-contrast sequence 13 weeks after initial symptom onset This scan depicts the pre-treatment cyst burden as it was performed one day prior to antiparasitic treatment. Overall cyst burden had increased from prior scans with some lesions showing increased edema and some with less. The lesions depicted are continuing to degenerate but remain in the colloidal or granular-nodular stages. The lesion in the anterior right frontal lobe (pane A) has decreased perilesional edema when compared to the prior scan. The anterior left frontal lobe lesion (pane B) seen on the prior scan is absent on this scan. New lesions are seen in the right and left parietal lobes (panes A and B). A significant amount of perilesional edema is seen surrounding a new lesion between the right and left frontal lobes (pane C) and is concerning for subarachnoid involvement. A new lesion is also depicted in the left hippocampus (pane D) with considerable perilesional edema. The posterior right midbrain lesion demonstrates a small decrease in perilesional edema (pane E).

**Figure 5:**
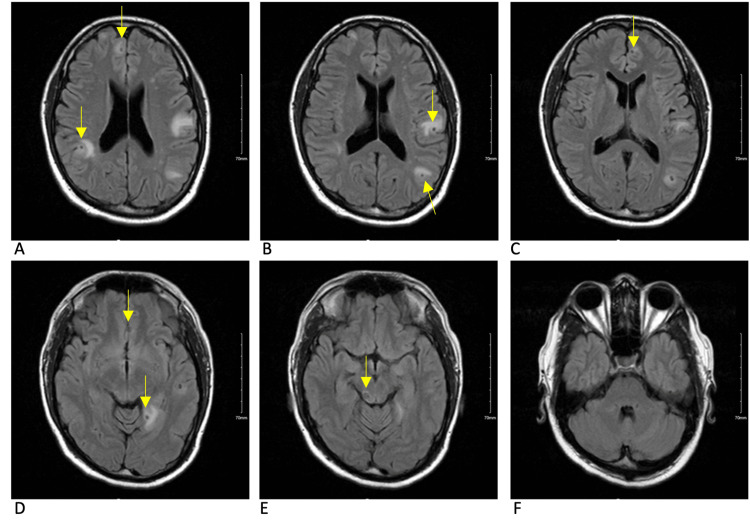
MRI brain T2 flair sequence 13 weeks after initial symptom onset Areas of inflammation that do not correlate to an obvious cyst in the T1 post-contrast sequence are seen in the left and right parietal lobes (panes A and B). The anterior right frontal lobe cyst has increased inflammation when compared to the prior scan (pane A). The anterior left frontal lobe cyst has resolved since the prior scan (pane B). The interhemispheric lesion (pane D) has minimal inflammation demonstrating the beginning of cyst degeneration. The left hippocampal lesion has significant inflammation associated with it (pane D). The right posterior midbrain lesion has slightly increased inflammation (pane E).

An MRI at 13 weeks from the initial presentation was performed and revealed a total of 16 lesions (increased from 11 total lesions just two weeks prior), many of which had enlarged and showed increased vasogenic edema and a new concern for subarachnoid involvement (Figures 4, 5). Although the patient had not experienced any further seizure activity since the initial presentation, the emergence of new lesions on serial imaging was concerning for severe, ongoing infection. At this juncture, a multispecialty discussion was held between neurology, infectious disease, ophthalmology, and radiology to coordinate treatment plans. The patient underwent a repeat ophthalmology examination that redemonstrated the absence of ocular involvement. Intraventricular NCC was also ruled out. The consensus favored that the benefits outweighed the risks of starting an antiparasitic regimen in the setting of increasing cyst burden.

**Figure 6:**
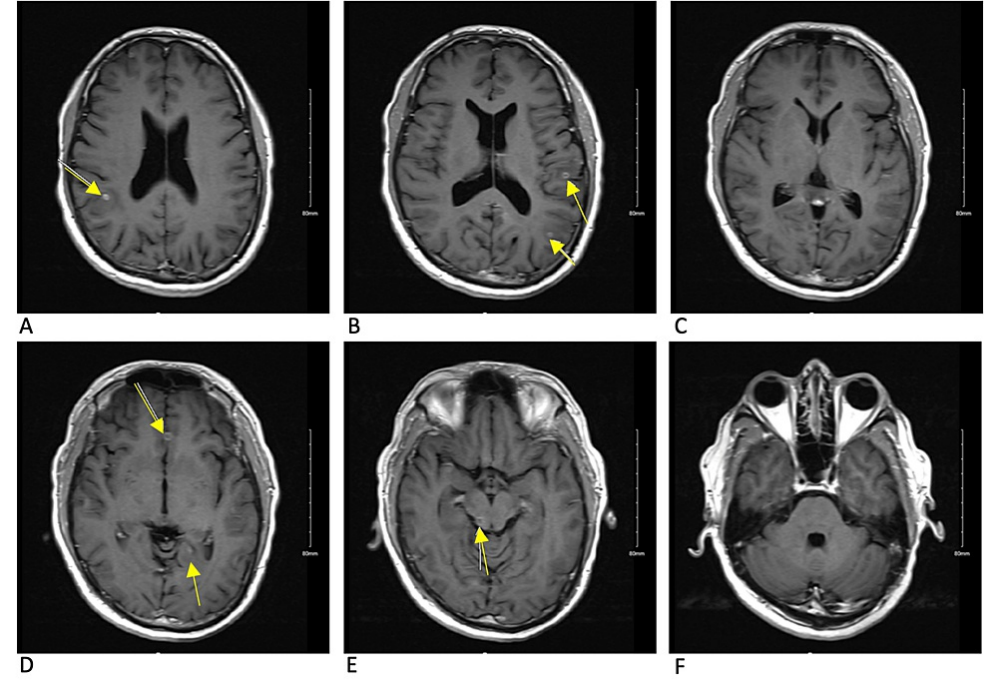
MRI brain T1 axial post-contrast sequence five days after initiation of dual antiparasitic therapy An overall decrease in the size of the ring-enhancing lesions is consistent with early response to antiparasitic treatment. Cysts are now progressing through the stages of degeneration and are mostly in the granular-nodular stage. The cysts seen in previous scans in the anterior frontal lobes are resolved (panes A and B). The cysts in the left and right parietal lobes have decreased perilesional edema (panes A and B). The interhemispheric, hippocampal, and right posterior midbrain cysts have decreased in size (panes D and E).

**Figure 7:**
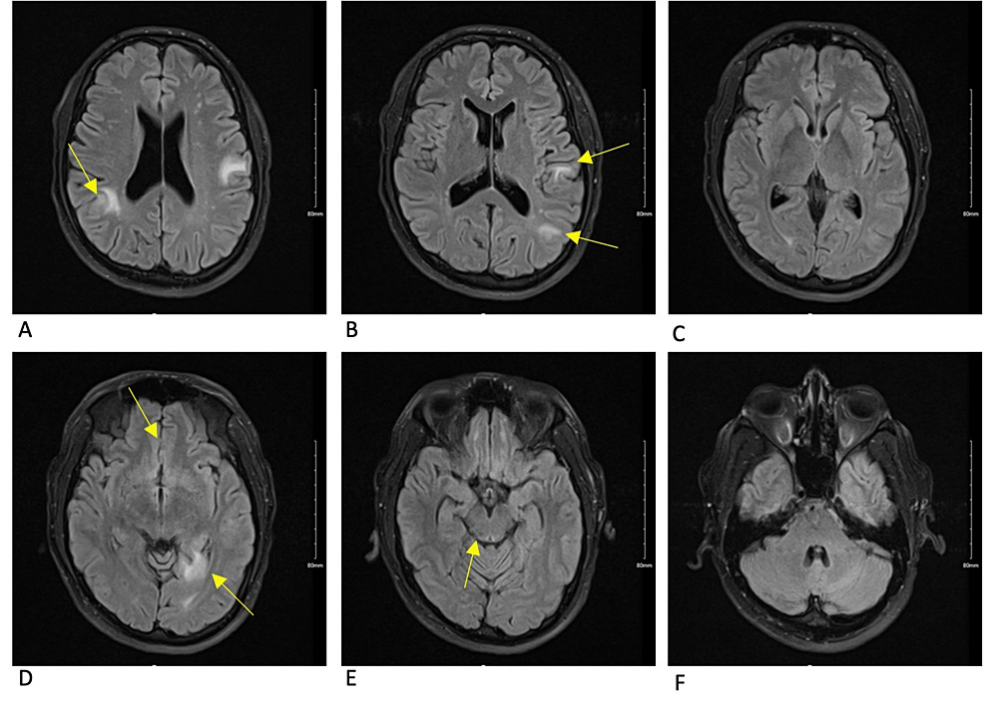
MRI brain T2 flair sequence five days after initiation of dual antiparasitic therapy An interval decrease in the size of the ring-enhancing lesions consistent with early response to antiparasitic treatment is demonstrated. The inflammation associated with the anterior right and left frontal lobes is resolved (panes A and B). The cysts in the left and right parietal lobes have decreased perilesional edema (panes A and B). The interhemispheric cyst and right posterior midbrain cyst have decreased in size (panes D and E). The inflammation associated with the hippocampal cyst has minimally increased (pane D).

The patient was admitted to the hospital for close observation with the initiation of antiparasitic treatment. The dexamethasone dose was increased from 1 mg per day to 6 mg per day for two days prior to hospital admission with a further increase to 8 mg per day while receiving antiparasitic treatment. The patient was started on albendazole 15 mg/kg/day divided into two doses and praziquantel 50 mg/kg/day divided into three doses while taking 8 mg dexamethasone daily. Repeat MRI on day five of admission (Figures 6, 7), revealed an interval decrease in the size of the ring-enhancing lesions consistent with early response to antiparasitic treatment. The patient did not experience any acute events and was discharged on day seven. The patient continued to take the same doses of albendazole, praziquantel, and dexamethasone for a total of 28 days with a steroid taper following.

**Figure 8:**
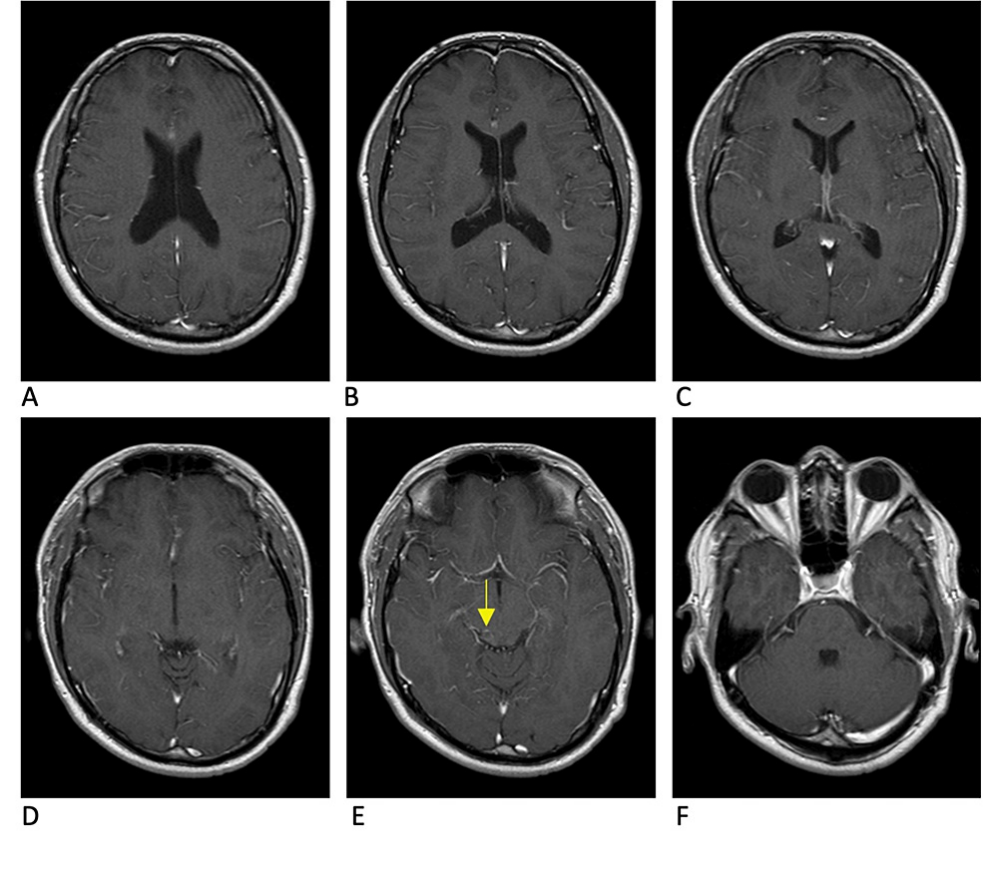
MRI brain T1 post-contrast sequence 31 weeks after dual antiparasitic treatment A significant reduction of cyst burden and edema is demonstrated. The lesion in the right posterior membrane is nearly resolved (pane E). All other cysts have resolved (panes A-D).

**Figure 9:**
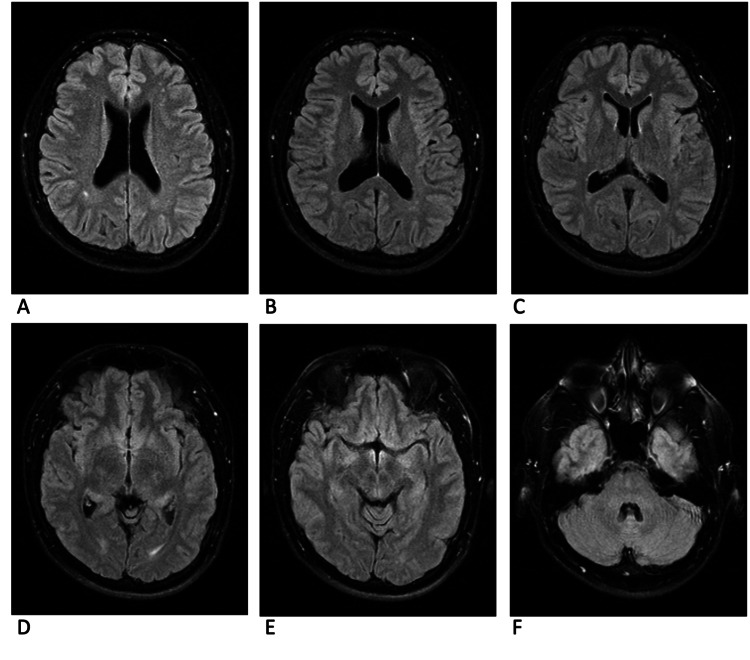
MRI brain T2 flair sequence 31 weeks after dual antiparasitic treatment Significant reduction of cyst inflammation is demonstrated (panes A-D). The lesion in the right posterior membrane is nearly resolved and has minimal inflammation (pane E).

MRI performed at 44 weeks from the initial presentation (31 weeks after initiation of antiparasitic treatment) (Figures 8, 9) demonstrated an overall decrease in inflammation of all lesions. The left parietal, left temporal, and brain stem lesions had nearly resolved. Importantly, there was no evidence of disease progression or new lesions. Oxcarbazepine was continued for seizure prophylaxis.

## Discussion

The patient described in this case report had both taeniasis (reported evidence of a tapeworm in his stool) and NCC. Co-infection with a live tapeworm and active NCC may be rare, even in endemic regions. One study showed that there is a greater prevalence of people who have seropositive cysticercosis in areas surrounding active tapeworm carriers (at home and close proximity). However, there is no increase in seizure prevalence in the active tapeworm carriers. This could be explained by the fact that tapeworms often die before the presentation of symptoms of NCC. Though the results of this study may not be generalizable to other populations, it suggests that it is less likely for a person to have an NCC-related seizure while also being a tapeworm carrier [9]. It is impossible to know when the patient in this case report was infected, as the latent period for NCC can be years. Endemic areas have an increased prevalence of contaminated pork as well as an increased risk of indirect transmission after eating food prepared by someone with taeniasis. It is likely that this patient developed NCC in an endemic area like India, but infection within the United States cannot be ruled out [1].

The treatment of NCC is multidimensional, but antiparasitics are invariably used, often combined with antiepileptics and corticosteroids. The balance between killing the cysts and limiting brain-damaging inflammation is delicate. Before antiparasitic treatment began in this patient, both ocular and intraventricular lesions were ruled out, as surgical removal is the preferred treatment for ventricular and ocular cysts. It is also important to note that tuberculosis and *Strongyloides* had to be ruled out before corticosteroid administration as both can reactivate under immunosuppression [8].

The initial approach to the patient, in this case, was to control and stabilize the parenchymal inflammation that had triggered his seizure before starting antiparasitic treatment, with particular concern for the risk of continued seizures and herniation from the midbrain lesion. Corticosteroids seem to have decreased inflammation around some of the cysts but not others, as demonstrated by Figures 2-5. Corticosteroids in this case also failed to prevent immune reactions to new cysts in the brain as demonstrated by new areas of inflammation on MRI at 13 weeks. With the potentially ongoing active spread of new cysts, the benefits of starting dual-antiparasitic treatment outweighed the risks of further inflammation. Specific guidelines and timelines in regard to the stabilization of edema with corticosteroids before starting treatment could be beneficial in the management of future cases.

Combined treatment with praziquantel and albendazole was chosen for our patient due to its proven efficacy for multiple parenchymal NCC. In addition, antiepileptics and corticosteroids were carefully incorporated. In the literature, treatment with antiparasitics plus antiepileptics has been shown to decrease the number of seizures and result in a greater number of resolved lesions than corticosteroids and antiepileptics alone [10]. These differences persist even without the use of antiepileptics, proving the necessity of antiparasitics in our patient’s treatment [11]. Cyclical use of albendazole has also been shown to reduce lesion load and seizure burden in patients with disseminated NCC [12]. Combined use of praziquantel and albendazole has been shown to be more effective in the destruction and resolution of brain cysticerci without increased adverse effects in comparison to albendazole alone. Partial seizures in the follow-up period are less common in patients with complete cyst resolution [13]. However, some patients may have a continuation of seizures after cyst resolution due to residual inflammation and scarring [3]. Our patient was continued on oxcarbazepine.

The above studies [10-13] demonstrate the importance of antiparasitic and antiepileptic drugs in the treatment of NCC, though control of the inflammatory process is perhaps equally as necessary. While antiparasitic therapy has been shown to reduce the long-term seizure burden, it can paradoxically result in greater inflammation that triggers symptoms, such as seizures, intracranial hypertension, and death [10-12,14-16]. Cyst degeneration and the release of antigens is the main driving factor for symptoms of NCC [3,14,15]. Cysts will eventually either calcify or resolve, though the timeline for this varies from three months to two years once the immune reaction begins. Calcified lesions can manifest with seizures and are a risk factor for long-term seizure recurrence [8]; however, it is more common to have seizures with degenerating lesions due to increased inflammation and edema [14]. The patient, in this case, has evidence of colloidal and granular-nodular cysts on MRI. The absence of calcified cysts indicates that this patient was acutely infected with viable cysts that had not completely progressed through the four stages of degeneration. We hypothesize that this patient had mostly vesicular cysts before taking albendazole, as he had no neurological symptoms. After the self-administered albendazole dose, the patient developed the presenting symptoms, leading us to believe that the albendazole dose disrupted the cysts’ immune tolerance and led to the beginning of their degeneration through the four stages.

Though corticosteroids are important for symptom control in the treatment of NCC, they are not sufficient for achieving long-term seizure freedom and cyst resolution in multi-parenchymal NCC [10,11]. Currently, there are no standard regimens for the administration of corticosteroids with antiparasitics for multi-lesional intraparenchymal NCC, though their use is quite prevalent and recommended [8,14].

There are multiple reports in endemic areas of treatment with praziquantel or albendazole for presumptive helminths as a regular “deworming” practice, which often results in the discovery of NCC. In these cases, seizures, malaise, headache, and focal neurologic deficits are precipitated by empiric antiparasitic treatment as in our patient. Concurrent corticosteroids and anti-epileptic drug administration likely would mitigate these adverse effects. In areas with a high burden of *T. solium*, it is common to have mass use of antiparasitics as a means of prophylaxis. In some of these areas, 10-20% of the general population have calcifications presumably from NCC, and 1-2% have asymptomatic live cysticerci [17-19]. It is plausible to perform serological testing and subsequent neuroimaging in endemic regions or in those with a travel history from these areas to avoid adverse outcomes of empiric antiparasitic treatment. In practice, this may not be reasonable due to cost and accessibility. Clinicians should be aware of the risks of “deworming” practices, especially in patients with risk factors for NCC.

## Conclusions

This case is unique in that our patient had both taeniasis and NCC and that a self-administered dose of albendazole is suspected of triggering the cerebral inflammation and the presenting symptoms. The balance between preventing further inflammation and administering anti-parasitic treatment is delicate. The presence of a lesion in the midbrain put our patient at high risk of obstructive hydrocephalus while lesions in the hippocampal region are highly epileptogenic. The delay of antiparasitic treatment was to avoid worsening symptoms, but this strategy led to the development of additional cysts. More education and awareness of the disease process and treatment protocol would be helpful in the future, as well as clear guidelines on the management of brainstem lesions and the administration of corticosteroids. Despite the low incidence of NCC in the United States, clinicians need to be aware of the possibility of co-infection of taeniasis and NCC, especially in patients who present with new-onset neurologic symptoms with a recent history of helminth discovery or antiparasitic treatment.
